# Poor Sleep Quality and Its Relationship with Individual Characteristics, Personal Experiences and Mental Health during the COVID-19 Pandemic

**DOI:** 10.3390/ijerph18116030

**Published:** 2021-06-03

**Authors:** Prerna Varma, Malisa Burge, Hailey Meaklim, Moira Junge, Melinda L. Jackson

**Affiliations:** School of Psychological Sciences, Monash University, Clayton, VIC 3800, Australia; malisa.burge@monash.edu (M.B.); hailey.meaklim1@monash.edu (H.M.); moira.junge@monash.edu (M.J.)

**Keywords:** sleep disturbances, stress, depression, anxiety, isolation, LIWC

## Abstract

While the COVID-19 has dramatically altered our lifestyle and sleep practices, the links between sleep, individual characteristics, personal experiences and mental health during the pandemic require further examination. This cross-sectional, multi-methods study examined differences in language used to describe personal experiences, and mental health, based on sleep quality during the early stages of the pandemic. *N* = 1745 participants (mean age 42.97 ± 14.46 years) from 63 countries responded to the survey. Sleep quality was assessed using the Pittsburgh Sleep Quality Index and mental health was examined using the Patient Health Questionnaire-9, the State Trait Anxiety Inventory, the Perceived Stress Scale and the UCLA-Loneliness Scale. Quantitative analysis of qualitative, language content of personal experiences was conducted using free-text responses and comments to a question on the survey. Almost 50% of the participants reported poor sleep quality, which was linked to a more negative emotional tone and greater mentions of money or finance related words. Good sleepers reported more positive emotional tone in their experiences. Greater reports of clinical state anxiety, moderate depression and moderate stress were observed in poor sleepers, even after accounting for demographics and pandemic-related factors such as loneliness, financial concerns and risk of contracting COVID-19 disease. Results from this study highlight an urgent need for sleep-related public health interventions. Practitioner education, sleep screening for those with mental health conditions, and encouraging people to adopt digital tools may help to reduce the burden of poor sleep on mental health. While the pandemic itself is a stressful and uncertain time, improving sleep can support positive emotion regulation, improving mood and consequential action.

## 1. Introduction

Good sleep quality plays a crucial role in maintaining positive well-being and mental health [1,2]. By contrast, sleep disturbances and related daytime dysfunction are a risk factor for reporting poor mental health [3,4,5,6,7]. As an example, sleep disturbances, such as insomnia, predict later onset of depression, anxiety, alcohol abuse and other psychotic illnesses [5,8,9]. The use of evidence-based treatments for sleep in individuals with psychiatric illness is beneficial for both sleep and mental health, even when the latter is not a primary target of the intervention [8,10,11]. Examining the interactions between sleep and mental health is now more important than ever, as the COVID-19 pandemic continues to disrupt daily life. While the negative effects of this pandemic on mental health are evident [12,13,14,15,16,17], their links with sleep disturbances require further investigation. Research investigating sleep disturbance may have translational benefits of improving sleep and reducing global mental health burden.

A number of studies have emerged since the pandemic began, indicating an overall decline in sleep quality and quantity [18,19,20,21,22]. These findings mirror previous pandemic studies (e.g., Ebola), where higher rates of poor sleep and greater presence of sleep disorders was observed in regions with higher infection rates [23,24]. Some studies have also shown improvements in sleep characteristics during the pandemic [25,26,27]. For example, Wright and colleagues, [27] reported an increase in total time in bed amongst university students during stay-at-home orders while Blume and colleagues [26] observed a reduction in social jetlag. Together, this demonstrates that the stay-at-home orders and other government restrictions may allow for more flexibility in sleep-wake routines, which in turn can increase sleep opportunity. However, in both studies, authors noted that despite improvements in sleep-wake flexibility and reduced social jet-lag, sleep quality during the pandemic still decreased. Other studies have also observed this decline in sleep quality and increase in insomnia symptoms during different stages of COVID-19 pandemic across different countries and populations [28,29,30,31,32]. Changes in lifestyle, pandemic stress, risk of contracting COVID-19 disease and financial distress may reduce sleep quality despite the availability of greater sleep opportunity. Similarly, studies have also documented the impact of the pandemic on mental health, including increased rates of depression, anxiety, stress and loneliness [15,18,33,34,35,36].

Based on pre-pandemic literature, poor sleep quality may be related to negative personal experiences and poor mental health during the pandemic. Poor sleep quality may explain why mental health continues to decline despite the increase in sleep opportunity. Significant changes to individual lifestyles and pandemic-related concerns (such as fears for personal or family health and anxieties over job stability) may increase stress and autonomic arousal, thus reducing sleep quality. In turn, these changes in sleep can exacerbate negative perception of events via an amplified amygdala response to stimuli [37], impacting emotion regulation and increasing stress. Together, this might explain the relationship between poor sleep and increased risk of depression and anxiety [7]. A positive feedback loop may exist, wherein increased presence of sleep problems and stressors progressively worsen each other through cyclic reinforcement, ultimately increasing the risk of experiencing anxiety and depression symptoms. As a result, it is important to understand stressors in people’s lived experiences, and how poor sleep quality may be associated with mental health during the pandemic.

While it is unclear what the ongoing impacts of the pandemic on mental health will be, our treatment responses need to adapt. Knowing the nuanced experiences felt during the pandemic can enrich our understanding of sleep and mental health, helping improve intervention strategies. Computerized text analysis systems like the Linguistic Inquiry and Word Count (LIWC) can be used to capture common qualitative narratives, quantitatively, in large datasets by decreasing labor and increasing objectivity [38]. The LIWC uses word processing and dictionaries to detect the frequency of word types across a variety of psychological categories, including emotional tone, social processes and thinking styles. In the context of the current study, a combination of trends in the language used to describe personal experiences and a summary of quantitative sleep and mental health characteristics can help provide a nuanced and detailed understanding of peoples experience during the pandemic, which may help inform future treatments and mitigation strategies.

Accordingly, this study aimed to; (a) determine the differences in language used to describe personal experiences of good sleepers compared to poor sleepers, and (b) explore associations between mental health and poor sleep. Specific demographic characteristics (such as age, gender and previously diagnosed mental health condition), and pandemic-related factors, such as loneliness, negative changes in financial situation (such as loss of job or income) and the risk of contracting the COVID-19 disease were also examined as covariates of associations between mental health and poor sleep.

## 2. Methods

This study presents data from the first wave of surveys disseminated globally between 9 April and 25 May 2020. The survey is a part of a longitudinal study examining changes in sleep and mental health across the pandemic. Some of the data from the baseline survey has been published previously [17]. The study was approved by Monash University Human Research Ethics Committee and conducted in accordance with Declaration of Helsinki. Participants were recruited via social media channels, including Facebook, Twitter and LinkedIn. Participants were given an opportunity to respond to the survey anonymously or provide their email at the start of the survey if they were interested in participating in the longitudinal waves of the study.

### 2.1. Measures

The survey included demographic items (age, sex, education, employment/student status, country of residence, ethnicity, marital status, and number and age of dependents), self-reported history of mental health diagnoses [“Have you been diagnosed with a mental health condition (either in the past or currently)?”], alcohol use (“Has the amount of alcohol, or the frequency with which you consume alcohol, changed since the COVID-19 crisis began? If yes, in what way?”), and questions related to stay at home orders [“Are you currently under lockdown or self-isolating due to the COVID-19 pandemic? (e.g., government orders for staying at home, working from home, limiting contact with others)?”], risk of contracting COVID-19 disease [“Are you at a high risk of COVID-19? (High risk refers to being over 70 years old; over 50 years old and of Aboriginal descent; pregnant; parent of a child under 12 months; under treatment for chronic health conditions, immune compromised.”)], financial situation (“Has the COVID-19 pandemic impacted your employment status?” “Has the COVID-19 pandemic impacted your financial situation?”), distress due to a change in financial situation (“How much distress has this change in financial status caused you?”), and type of change in their sleep patterns [“Have your sleep patterns changed since the start of the pandemic?”, “If yes, how have your sleep patterns changed?”, and “Do you think your sleep-wake routine (e.g., bed-time and get-up time) is more consistent with your personal preference or “body clock”, than before the lock down/self-isolation/quarantine period? (e.g., night owl able to go to bed later as you don’t have to get up early for work)]. Participants were also asked to self-report their sleep quality just before the pandemic in comparison to their sleep during the data collection period. An optional, free-text question on comments regarding sleep and experiences during the pandemic was also included (“Are there any comments you would like to make about your sleep or changes to your life during COVID-19 pandemic?”).

The Pittsburgh Sleep Quality Index (PSQI) was used as a validated measure of self-reported sleep quality. The questionnaire has seven domains: subjective sleep quality; sleep latency; habitual sleep efficiency; sleep disturbances; use of sleep medication; and daytime dysfunction (e.g., sleepiness, lack of enthusiasm to carry on daily activities). The seven domains are scored from 0–3, with 3 indicating sleep disturbance in a particular domain, with a total score of 21. PSQI has high reliability (Cronbach’s alpha of 0.85) and validity [39] and is one of the primary measure used in assessment of sleep health. To classify participants as “good” or “poor” sleepers, PSQI ≥ 8 was used as a cut-off [39,40,41], which is a validated cut-off point used in other studies. PSQI score of eight was also the median score for this cohort.

The Patient Health Questionnaire (PHQ-9) was used to examine depressive symptoms over the last 2 weeks. PHQ-9 scores range from 0 to 27, with scores ≥10 recommended as a cut-off for moderate depression. The scale has excellent reliability (Cronbach’s alpha of 0.89) and high construct validity, making it one of the primary instruments used for depression screening [42,43]. Anxiety was assessed using the well validated and reliable 6-item State Trait Anxiety Inventory (STAI) [44]. Six items related to anxiety were rated on a 4-point scale, with higher scores indicating more anxiety. A score of 40 or more was used as the cut-off for clinical anxiety. The Perceived Stress Scale (PSS) was used to assess current stress state and has excellent psychometric properties, with scores above 14 suggesting moderate to severe levels of stress [45,46]. Loneliness was examined using the UCLA-Loneliness scale short form (UCLA-LS), which has three questions that measure loneliness and social isolation.

### 2.2. Data Analysis

All quantitative data except for the LIWC group differences were analysed using IBM SPSS (version 26.0, IBM, Armonk, NY, USA). PSQI cut-off of eight or more was used an indicator of poor sleep and participants were accordingly divided into “good” or “poor” sleepers based on their PSQI scores. Chi-square analysis were used to examine differences in demographic characteristics among good and poor sleepers.

To examine differences in the language participants used to describe their personal experiences during the pandemic, content analysis of language was performed using the Linguistic Inquiry and Word Count (LIWC) software [38] and further analyzed using R (version 3.6.3) [47]. The LIWC dictionary includes psychologically validated categories of words (e.g., anxiety words could include “nervous”, “afraid”, or “tense”). Frequency of a word category is measured by the percentage of occurrence of a particular word category within a piece of text. In doing so, the LIWC allows the gathering of quantitative summary data from qualitative writing. Prior to the LIWC analysis, data were cleaned for missing text (*n_missing_ =* 431) in comments (*n*_remaining_ = 1103). Non-parametric Mann-Whitney U tests were then used to examine content differences on LIWC word categories between poor sleepers and good sleepers. The LIWC word categories of interest included the 33 sub-categories of psychological processes, including affective processes, biological processes, drives, perceptual processes, personal concerns, and social processes [48]. To control for alpha inflation, all significance values were compared to their critical value after a Benjamini-Hochberg correction [49]. Prior to conducting LIWC analysis, a logistic regression (in R version 3.6.3) was also performed on the demographics to identify what factors influenced the likelihood of providing a free-text comment (used in the LIWC analysis). The most frequently observed category was used as the dummy category (i.e., female, bad sleepers, Australia-New Zealand, in lockdown, bachelor’s degree or higher, and employed full-time).

To examine the incidence of poor sleep, clinical levels of state anxiety, moderate depressive symptoms and moderate stress among participants in this sample, cut-offs for each scale (PSQI, STAI, PHQ-9 and PSS) were used to create dichotomous variables. Associations between these dichotomous variables and poor sleep were analyzed using binary logistic regression. Two models were examined. The first model included age and sex as covariates along with poor sleep. The second model examined prior diagnosis of a mental health condition, and pandemic-related factors such as, isolation, negative change in financial situation and risk of contracting COVID-19 disease as covariates along with poor sleep. Odds ratios (with 95% CI) were calculated to examine prevalence of anxiety, depression and anxiety in individuals with poor sleep.

## 3. Results

### 3.1. Participant Characteristics

A total of 2555 participants responded to the survey. Data from 1745 respondents (who provided complete response to PSQI) were included in the study. Based on PSQI scores, 47% of the participants reported poor sleep quality. Within the PSQI subscales, 55% percent of respondents reported having difficulty falling asleep at least 2 nights per week. General sleep disturbances, such as feeling hot or cold at night, having pain or experiencing nightmares (at least 2 times per week) were reported by 38% of the participants. When asked to compare their sleep quality during the pandemic with their sleep pre-pandemic, 20% of the participants reported improved sleep, whereas 57% reported poorer sleep compared to their sleep pre-pandemic. Overall, 74% of participants reported that their sleep patterns had changed since the start of COVID-19 pandemic. Of these, the majority of respondents reported that they were sleeping and waking up later than their usual bedtimes/waketimes before the pandemic began (*n* = 361, 22% of the sub-sample). Forty-three percent of the sample (*n* = 685) reported that their sleep patterns were now more in sync with their body clock (i.e., their personal preferences for wake and sleep) compared to before the pandemic.

Participant characteristics across good and poor sleepers are reported in Table 1. Differences in sleep quality and mental health based on participants’ country of residence have been reported previously [17]. Broadly, small, non-significant differences were observed between countries with the highest response rate (Australia, India, United Kingdom, South Africa and United States of America).

More than 90% of the participants were in a government-imposed lockdown at the time of completing the survey, broadly defined as stay-at-home orders, or going out only for shopping essential items or for work when such work could not be performed from home. Poor sleepers reported higher levels of unemployment and disability than good sleepers. Poor sleepers also reported significant increase in their alcohol consumption since the start of the pandemic as opposed to good sleepers.

### 3.2. Poor Sleep and Personal Experiences

Since the question related to personal experiences was optional, a logistic regression was conducted to determine which demographic groups were more likely to respond (Supplementary Table S1). Specifically, age, poor sleep quality, country of residence in Asia or Africa was associated with greater odds of responding to the question. Gender, lockdown status and education were not related to the likelihood of providing a free-text answer.

Next, differences in the language used by participants to describe their personal experiences based on whether they were good or poor sleepers were analyzed using LIWC. Of the 33 LIWC categories relating to psychological processes, eight word-categories demonstrated significant differences between poor and good sleepers after Benjamini-Hochberg correction of critical values. Between poor sleepers and good sleepers’ significant differences were demonstrated in their emotionality (i.e., emotional tone), social processes, and money-related words (see Table 2). Notably, poorer sleepers used more negative emotion and anxiety related words in their comments. Examples of these comments from poor sleepers:

“*Feeling anxious, either can’t sleep or sleep 12 h...*” and,

“*I cannot find a job because of the situation, that has caused me a lot of distress, anxiety, and therefore I’m taking pills for sleep*”.

Additionally, poorer sleepers had a higher frequency of words relating to money, which may result from financial concerns as reflected in the following comment:

“*The insecurity of income, oppressive and extended lockdown of undetermined duration and potential of violent uprisings due to hunger as well as worrying about unpaid rental and utility bills is causing extreme anxiety.*”

Conversely, good sleepers used more words relating to positive affect and social processes, including family and friends. One good sleeper commented:

“*Able to sleep properly may be due to spending more time with family.*”

### 3.3. Links between Poor Sleep and the Risk of Poor Mental Health

Both regression models revealed greater odds of clinical state anxiety, moderate depression and moderate stress among participants with poor sleep (Table 3). In particular, Model 1 showed that poor sleep quality was associated with up to four times higher odds of reporting anxiety, depression and stress.

Age was a significant covariate, with lower odds of poor mental health observed with increasing age. Pandemic-related factors, such as loneliness and negative change in financial status were associated with higher odds of reporting depression and anxiety. However, even after accounting for pandemic-related factors and prior diagnosis of a mental health condition, the risk of reporting depression and or anxiety was three times higher in poor sleepers [OR = 3.0 (CI = 2.37−4.27), OR = 3.01 (CI = 2.37−3.84), respectively] as opposed to good sleepers.

Like depression and anxiety, greater loneliness and a negative change in financial situation were associated with higher reports of stress. Interestingly, individuals who self-reported as not at risk of contracting COVID-19 were more stressed that those who were at-risk.

## 4. Discussion

This study aimed to examine the links between sleep, differences in language used to describe personal experiences and mental health during the early stages of the COVID-19 pandemic. Overall, poor sleep quality was observed in the current sample, with almost half of the participants scoring above the cut-off of 8 on the PSQI. Similar to past research [12,13,50,51,52], elevated levels of stress, anxiety and depression were reported in this study.

Additionally, the LIWC language analysis revealed that individuals with good sleep had a more positive emotional tone when reporting on their personal experiences, consistent with our findings on validated measures of mental health. Conversely, poor sleepers used more negative emotional tone words, displayed more anxiety, frequently mentioned finance related words as opposed to words related to social interactions or processes. This was also observed in validated questionnaires, where poor sleepers were more likely to indicate changes in their financial status, increased alcohol consumption, and were at least three times more likely to report depression, anxiety and stress. In particular, poor sleep was associated with the highest odds of reporting clinical state anxiety, moderate depression and moderate levels of stress, even after controlling for demographic and pandemic-related factors. Together, this suggests that independent of pandemic-related factors or demographic differences, sleep is important for overall wellbeing.

While previous studies have reported an increase in sleep-wake flexibility and greater sleep opportunity [20,45,53,54], the current study observed poorer sleep quality and an increase in insomnia-related symptoms. This may be explained by a number of factors. Firstly, the increased mental health burden due to isolation, lockdowns and change in lifestyles may have heightened arousal, increasing both stress and poor sleep. Secondly, time gained from not needing to commute due to stay-at-home orders, may be used for work and not for sleep [55]. As a result, overall sleep quality may not have changed. Further, average bed-and wake-times have shifted during the pandemic [56], potentially leading to less sleep-wake regularity and decreased sleep quality. Finally, lockdowns, restriction in outdoor activities and excessive use of light-emitting screens may increase circadian misalignment due to lack of exercise and reduced natural light exposure. Pre-bed light hygiene like frequent phone use before bedtime, may have worsened sleep quality [57]. Interestingly, in the current study, only 25% of the participants were receiving more than 30 min of daily sunlight exposure, and individuals with poor sleep quality reported increased phone usage at night (in comparison to before the pandemic). This may involve ‘doomscrolling’, or increased consumption of negative content online, which can heighten arousal before bed and potentially affect both sleep and mental health. As an additional consequence, increased pre-bed phone usage can also disrupt melatonin secretion (i.e., major sleep promoting hormone in humans), resulting in delayed sleep timing or increased difficulties with sleep initiation [58].

### 4.1. Poor Sleep and Personal Experiences

A novel aspect of this study was the qualitative synthesis of personal experiences of people experiencing good and poor sleep quality. Results revealed that more poor sleepers reported increased alcohol use, greater phone use and more loneliness. This suggests the precarious impact of a pandemic on lifestyles and overall wellbeing. Several themes also emerged from the language content analysis of comments that participants made about their sleep and mental health. For instance, the content from good sleepers had a more positive emotional tone. When describing their personal experiences, good sleepers also made more frequent use of words around social processes (such as “ally” or “friends”), which may indicate the protective role that social interactions can play in improving mental health and therefore sleep. Good sleepers used more family-related words, such as “husband” or “child” as opposed to poor sleepers. Because a full qualitative analysis was unable to be conducted (due to the large sample size), the context in which family-related words were used is not entirely clear. This could be positive (e.g., enjoying time with family) or negative (e.g., worrying about family). However, the current questionnaire findings of fewer good sleepers reporting feelings of isolation suggests that this group likely has a stronger support network, which may be protective against sleep disturbances. Additionally, the results are similar to another COVID-19 study [59], which suggested that increased sleep quality is associated with social capital, a concept that includes social trust, belongingness and participation. Potentially, individuals with more social capital may experience less loneliness and isolation, which could act as a buffer against poor sleep.

By contrast, poor sleepers used more negative emotional tone words such as “hate,” “strange” and “isolation” when compared to good sleepers. Interestingly, the LIWC negative emotional tone category has been previously used to index for stress and depression [60]. As a result, the increased use of negative emotional tone may reflect greater levels of stress and depression experienced by poor sleepers. Poor sleepers also made more mentions of finance-related words such as “cash”, “money”, and “owe”. The accompaniment of higher finance related words and negative emotional tone might indicate that poor sleepers are experiencing more financial distress. This was also reflected in the quantitative analysis, wherein a greater proportion of poor sleepers indicated negative financial changes. Regression models revealed that these financial changes, related to loss of job or income accounted for increased risk of reporting depression, anxiety and stress. While we do not know whether any individuals were receiving some form of government financial support, it is possible that any support could have positive implications for their sleep and mental health.

### 4.2. Links between Poor Sleep and the Risk of Reporting Anxiety, Depression or Stress

Results from the study demonstrate that poor sleepers had greater incidence of reporting anxiety, depression and anxiety. These associations were observed even when major demographic and pandemic-related concerns were accounted for results revealed that poor sleepers had more than three times higher odds of reporting clinical anxiety and moderate depression, and almost three times higher odds of reporting moderate levels of stress. This not only highlights the well-established links between sleep and mental health, but also shows that while changes to lifestyle during the pandemic may be related to mental health, addressing sleep could potentially help mitigate some of the negative effects.

Given the cross-sectional nature of this analysis, it is difficult to determine the direction of this association. It is plausible that heightened anxiety and stress experienced as a result of the pandemic preceded disturbances in sleep. Conversely, changes in sleep may have occurred first, exacerbating daytime mood and stress symptoms [61]. Alternatively, both sleep and psychological distress may have emerged concurrently. Ongoing longitudinal assessments in the cohort will help us expand on these associations further, giving us an opportunity to examine causal effects between sleep and mental health.

### 4.3. Sustained Public Health Interventions for Improving Sleep Quality

As shown in this study, poor sleepers had more negative personal experiences, greater stress and higher reports of depression and anxiety, independent of personal and pandemic-related factors. Considering that mood and anxiety disorders share bidirectional associations with sleep abnormalities [5], treating sleep can be a cost-effective, efficacious way of improving overall wellbeing. This can reduce the perpetuating effects of poor sleep on mental health. Previous studies have shown that treatments for sleep can also improve anxiety and depression symptoms [11,62,63], including both face-to-face and digital Cognitive Behavior Therapy for Insomnia (CBT-I and dCBT-I, respectively). There is also growing evidence of the efficacy of self-help tools, such as mindfulness apps, to treat subclinical and clinical symptoms of sleep disturbances [64]. While treating sleep may not directly address the negative experiences of a stressful, uncertain pandemic, it can help reduce their propensity and improve individual reaction to the events. For instance, brain regions responsible for rapid eye movement (REM) sleep (such as the amygdala) mediate the stress response [65]. These brain regions also show convergence towards emotional reactivity and consequential action [66], which can prepare individuals for responding to stressful events such as the COVID-19 pandemic. For example, a recent study reported that individuals who received evidence based digital treatment for insomnia had greater resilience and better mental health during the pandemic than those who did not [62]. Given that there is a dearth of consistent sleep training in healthcare professionals such as psychologists, general practitioners and pharmacists [67], upskilling the workforce in sleep treatment delivery, or increasing awareness and education about bidirectional associations between sleep and mental health, may improve overall mental health within communities.

Lastly, providing sustained and consistent public health messages on sleep can also be the key to improving mental health outcomes. If research during this pandemic is any indication, people are experiencing a cluster of poor sleep and mental health issues, which can become chronic if left unaddressed. Public health message should be aimed at increasing the uptake of good sleep practices, which include modifiable behaviours. Given that poor sleepers had greater phone and alcohol usage, helping people create boundaries around phone usage at bedtime and alcohol consumption may be helpful. Additionally, the task force of the European CBT-I Academy suggests practices such as keeping a regular bed-time and wake-time (i.e., bringing more structure to sleep routines), having time to de-stress and reflect, and greater exposure to sunlight [26] for sleep retraining. Encouraging individuals to adopt these broader sleep practices can help improve sleep within the community.

## 5. Strengths and Limitations

The study derives its strength from recruiting participants globally, representing different countries, ethnicities, age-groups and communities. It also uses a clear, well-validated measure of sleep quality and provides novel documentation of personal experiences during the pandemic. Furthermore, the study accounts for links between sleep characteristics and mental health after controlling for personal and pandemic-related factors, which provides greater understanding of how the pandemic itself may be related to poor mental health and what role sleep may play when it comes to anxiety, depression and stress.

However, results from this study should not be over-stated. While there is significant convergence between sleep disturbances and negative mental health experiences, the data presented here is cross-sectional and cause-effect associations cannot be determined. Individuals with sleep or mental health issues may have been more inclined to respond to the survey, which may have led to bias. However, it must be noted that the study did not specifically set-out to recruit participants with sleep or mental health concerns, instead it broadly framed advertisements as questions about “sleep and mental health” during the pandemic. Further, the study compared participants who slept poorly versus those who slept well, which may help reduce any survey response bias.

There were certain demographic groups that were more likely to respond to the question on personal experiences. While education itself was not a factor associated with likelihood of response, cross-cultural differences in the use of language may have an impact on the results. Cross-cultural studies comparing interview transcripts from Czech Republic, Poland, Turkey and Germany (translated to English) in differences of language about traumatic events suggests some differences in the use of LIWC categories such as cognition, but not affective processes that were used in this study [68]. Across cultures, differences in pronoun-use, use of perceptual and social language exist [69]. However, similar to Freitag et al., our study did not find any significant differences in use of affective processes across countries. Together this suggests that affective processes may be more robust to differences in culture and may be more reliable as a cross-cultural linguistic feature compared to other word types. Regardless, differences in language between good sleepers and bad sleepers should be interpreted cautiously.

Building on the evidence from this growing body of evidence of the impact of the pandemic on both sleep and mental health is crucial. Since longitudinal analysis have shown that mental health may evolve during this period [70], more multi-wave studies are required to examine how both sleep disturbances and mental health symptoms may change, especially as different countries go in and out of lockdowns. Whether or not these conditions disproportionately affect certain ethnicities, nations, workers or individuals with pre-existing mental health conditions needs to be understood further.

## 6. Conclusions

Results from the first-wave of our global, longitudinal survey indicate that poor sleep quality is common during the pandemic, and is associated with a 2–3 times increase risk of reporting state anxiety, moderate depression and stress in comparison to good sleepers. Specific factors such as prior diagnosis of mental health condition, financial changes and loneliness, along with sleep characteristics were linked to poor mental health, suggesting that while sleep problems increase the odds of experiencing poor mental health, not all individuals are impacted by the pandemic in the same way. People experiencing distress due to changes to their financial situation and employment, and individuals with pre-existing mental health conditions are some of the vulnerable groups in our community who reported poorer sleep and mental health outcomes, and whom may need additional and ongoing support. Sustained public health messaging on improved sleep practices and increased dissemination and accessibility of self-help tools to aid sleep and mental health are crucial to improve sleep and reduce psychological distress during these uncertain times.

## Figures and Tables

**Table 1:** Participant characteristics (*N* = 1745).

Demographics	Mean ± SD and Percentage	Good Sleepers *n* = 925	Poor Sleepers *n* = 820	*p*-Value
Age	42.97 ± 14.46y (18–82) ^a^			
Gender (*n* = 1727)				0.06
Males	554 (31.7%)	291 (31.5%)	263 (33%)	
Females	1158 (66.4%)	629 (68%)	527 (65.9%)	
Self-describe	15	5	9	
Education (*n* = 1625)				<0.01
Bachelor’s degree or higher	1322 (81.54%)	736 (85%)	590 (77%)	
Vocational college or trade diploma	265	56	86	
High school	122	58	64	
Less than high school	19	5	14	
Other	25	10	15	
Employment status (*n* = 1625)		(*n* = 856)	(*n* = 769)	<0.001
Employed full-time	819 (50.2%)	452 (52.8%)	365 (47%)	
Self-employed	182 (11.2%)	99 (11.5%)	82 (10%)	
Employed part-time	143	76	67	
Retired	114	50	50	
Unemployed	72	35	47	
Disabled	13	2	11	
Student	150	95	55	
Other (casual part time or full time/stay at home parent)	138	52	86	
In lockdown	1582 (90%)			
Working from home (*n* = 1505)	1003 (67%)	582 (72%)	421 (60%)	<0.001
More than one dependent	709 (43%)	361 (43%)	348 (44%)	0.24
Self-reported sunlight exposure > 30 min daily	427 (27%)	231 (26%)	196 (22%)	0.82
Increase in phone usage during the pandemic	767 (41%)	369 (43%)	398 (51%)	0.01
Increase in alcohol usage *(if consumed)* during the pandemic (*n* = 1303)	527 (40%)	236 (36%)	291 (44%)	<0.001
Negative financial change due to the pandemic	977 (55%)	390 (40%)	587 (60%)	<0.001
At risk of contracting COVID ^b^	300 (18%)	111 (37%)	189 (63%)	<0.001
Diagnosed with a mental health condition before the pandemic	473 (29%)	184 (21%)	289 (36%)	<0.001
Sleep duration (in hours)	6.59 ± 1.46 (0–11) ^a^	7.31 ± 1.14	5.76 ± 1.46	<0.001
Perceived stress (PSS > 14) ^c^	1273 (73%) ^b^	646 (83.8%) ^b^	556 (64.7%) ^b^	<0.001
State anxiety (STAI > 40) ^c^	963 (59.0%) ^b^	400 (46.5%) ^b^	563 (73.0%) ^b^	<0.001
Depression (PHQ-9 > 10) ^c^	578 (35%) ^b^	185 (21.5%) ^b^	392 (50.7%) ^b^	<0.001

PHQ-9—Patient Health Questionnaire—9, PSQI—Pittsburgh Sleep Quality Index, PSS—Perceived Stress Scale, STAI—State-trait Anxiety Inventory (state subscale). Significance values obtained from independent samples t-test (for age and sleep duration), and chi-square (for all variables except age and sleep duration). ^a^ represents range. ^b^ total sample size was 1631. ^c^ cut-offs for poor sleep, moderate-to-severe levels of stress, clinical state anxiety, moderate-to-severe depression symptoms for PSQI, PSS, STAI and PHQ respectively.

**Table 2:** Differences between poor sleepers (*n* = 601) and good sleepers (*n* = 501) for LIWC categories based on the language used by participants to describe their experiences during the pandemic.

Word Type	Example Words	Poor Sleepers ^1^ Mean (SD)	Good Sleepers ^1^ Mean (SD)	*p*-Value	B-H Critical Value ^3^
Overall emotional Tone ^2^		21.18 (24.39)	32.12 (30.92)	<0.001 *	0.001 *
Negative Emotion	hate, worthless, enemy	7.71 (15.03)	4.27 (10.68)	<0.001 *	0.003 *
Positive Emotion	happy, pretty, good	1.44 (4.70)	3.04 (7.91)	<0.001 *	0.004 *
Anxiety	nervous, afraid, tense	3.48 (10.73)	1.69 (6.79)	<0.001 *	0.006 *
Money	audit, cash, owe	0.85 (3.95)	0.25 (1.50)	0.001 *	0.007 *
Social Processes	talk, us, friend	1.13 (3.75)	2.34 (6.59)	0.001 *	0.008 *
Affiliation	ally, friend, social	0.49 (2.83)	1.04 (3.94)	0.002 *	0.010 *
Family	mom, brother, cousin	0.24 (1.53)	0.76 (3.86)	0.004 *	0.011 *

^1^ Poor-sleepers and good sleepers based on cut-offs on Pittsburgh Sleep Quality Index (PSQI), where individuals with sleep quality score of PSQI < 8 were determined as ‘good’ sleepers and individuals with PSQI ≥ 8 were determined as ‘poor’ sleepers. ^2^ Emotional tone, as identified by language analysis of free-text comments by participants. For instance, positive emotional tone refers to using more upbeat language. Overall emotional tone includes both negative and positive emotions. Social processes include words that indicate social relationships like family, friends etc. ^3^ Benjamin-Hochberg critical value for false positive rate. * significant at respective critical value after applying Benjamini-Hochberg correction.

**Table 3:** Binary logistic regression examining the links of personal characteristics, pandemic and sleep-related factors with the severity of anxiety, depression and stress symptoms (based on cut-offs of each scale).

Factor	Anxiety Symptoms ^5^ OR (98% CI) ^§^		Depression Symptoms ^5^ OR (98% CI) ^§^		Stress Symptoms ^5^ OR (98% CI) ^§^	
	Model 1	Model 2	Model 1	Model 2	Model 1	Model 2
**Age**	0.96 (0.95–0.97) ***	0.97 (0.96–0.98) ***	0.97 (0.96–0.98) ***	0.98 (0.97–0.99) ***	0.97 (0.95–0.98) ***	0.98 (0.97–0.99) ***
**Sex**	0.81 (0.65–1.01)	0.81 (0.64–1.03)	0.83 (0.66–1.01)	0.81 (0.68–1.12)	0.76 (0.55–1.0)	0.76 (0.54–1.06)
**Prior diagnosis of a mental health condition**		1.29 (0.99–1.68)		1.88 (1.45–2.44) ***		1.86 (1.34–2.58) ***
**UCLA-LS ^1^**		1.40 (1.31–1.50) ***		1.41 (1.31–1.52) ***		1.46 (1.34–1.59) ***
**Negative change in financial status ^2^**		1.22 (1.07–1.39) **		1.30 (1.14–1.48) ***		1.47 (1.25–1.72) **
**Not at risk of developing severe symptoms ^3^**		1.02 (0.75–1.40)		0.78 (0.56–1.07)		1.78 (1.13–2.79) *
**Poor Sleep ^4^**	3.71 (2.96–4.66) ***	3.01 (2.37–3.84) ***	4.44 (3.52–5.59) ***	3.34 (2.61–4.27) ***	4.03 (2.91–5.57) ***	2.95 (2.08–4.17) ***

^1^ UCLA-Loneliness Scale-short form (UCLA-LS). ^2^ Change in financial status due to the pandemic. ^3^ Not at risk of developing severe symptoms of COVID-19, self-reported by the participants. ^4^ Poor sleep defined as a score >8 on Pittsburgh Sleep Quality Index (PSQI) ^5^ Anxiety symptoms refers to state anxiety, measured by State Trait Anxiety Inventory (STAI). Depression symptoms was measured using Patient Health Questionnaire-9 (PHQ-9) and stress symptoms was measured using Perceived Stress Scale (PSS). Each variable was re-coded dichotomously, representing clinical state anxiety (STAI score >40), moderate-to-severe depression (PHQ-9 >10), and moderate-to-severe stress (PSS >14). ὰ Participants with incomplete/missed responses to any of the measures listed above were not included. ^§^ represents adjusted Odds Ratio. Model 1 (*n* = 1438) adjusted for age and sex. Model 2 (*n* = 1433) adjusted for age, sex, loneliness, prior mental health diagnosis, change in financial status due to COVID-19 pandemic, and at-risk of contracting COVID-19 disease. * *p* < 0.05, ** *p* < 0.01, *** *p* < 0.001.

## Data Availability

Data availability is currently restricted due to ongoing data collection and to maintain participant privacy.

## Supplementary Materials

The following are available online at https://www.mdpi.com/article/10.3390/ijerph18116030/s1, Table S1: Logistic regression of demographics predicting answering of free-text question used in LIWC analysis.
