# Supplementary material for: Poor Sleep Quality and Its Relationship with Individual Characteristics, Personal Experiences and Mental Health during the COVID-19 Pandemic
Source: Int J Environ Res Public Health. 2021 Jun 3;18(11):6030. doi: 10.3390/ijerph18116030 (PMC8200012; doi:10.3390/ijerph18116030)
Supplement: Supplementary file 1 [file ijerph-18-06030-s001.zip › ijerph-1234461-supplementary.pdf]

Table S1. Logistic regression of demographics predicting answering of free-text question used in LIWC analysis.

Demographics	Estimate	Standard Error	*z* Score	*p*-Value
(Intercept)	0.12	0.28	0.42	0.675
**Age**	0.02	0.01	3.85	<0.001
**Gender**				
Male	0.23	0.15	1.58	0.114
Self-describe	14.78	413.89	0.04	0.972
**PSQI Good Sleepers**	−0.32	0.13	−2.54	0.011
**Region**				
Africa	1.02	0.30	3.44	0.001
Asia	0.48	0.19	2.48	0.013
Europe	0.02	0.16	0.13	0.895
North America	−0.24	0.25	−0.95	0.342
South America	−0.57	0.70	−0.82	0.414
**Not in Lockdown**	0.13	0.24	0.54	0.589
**Employment**				
Disabled	14.21	540.46	0.03	0.979
Part-time	−0.02	0.21	−0.09	0.926
Other	−0.23	0.41	−0.56	0.574
Retired	0.31	0.34	0.91	0.363
Self-employed	−0.68	0.20	−3.45	0.001
Student	−0.05	0.23	−0.22	0.826
Unemployed	−0.10	0.26	−0.40	0.692
**Education**				
Less than high school	−0.58	0.54	−1.08	0.282
Other	−0.10	0.24	−0.42	0.675
Vocational college or trade diploma	0.09	0.24	0.36	0.719

n = 1438 cases used in estimation; McFadden's Pseudo R-squared = 0.165; AIC = 1613.3; multiple comparisons correction: None. PSQI = Pittsburgh Sleep Quality Index
